# Robotic monitoring of forests: a dataset from the EU habitat 9210* in the Tuscan Apennines (central Italy)

**DOI:** 10.1038/s41597-023-02763-2

**Published:** 2023-12-01

**Authors:** Mathew J. Pollayil, Franco Angelini, Leopoldo de Simone, Emanuele Fanfarillo, Tiberio Fiaschi, Simona Maccherini, Claudia Angiolini, Manolo Garabini

**Affiliations:** 1 Centro di Ricerca “Enrico Piaggio”, and Dipartimento di Ingegneria dell’Informazione, Universitá di Pisa, Largo Lucio Lazzarino 1, 56122 Pisa, Italy; 2 Department of Life Sciences, University of Siena, Via Mattioli, 4, 53100 Siena, Italy; 3 NBFC, National Biodiversity Future Center, 90133 Palermo, Italy

**Keywords:** Electrical and electronic engineering, Plant ecology, Biodiversity

## Abstract

Effective monitoring of habitats is crucial for their preservation. As the impact of anthropic activities on natural habitats increases, accurate and up-to-date information on the state of ecosystems has become imperative. This paper presents a new dataset collected from the forests located in the Tuscan Apennines (Italy) using the ANYmal robot. The dataset provides information regarding the structure and composition of the EU priority habitat 9210*. The dataset, which is publicly available through a Zenodo repository, includes photos, videos, and point clouds of the environment. This dataset is a valuable resource for the scientific community working in the field of forest ecology and conservation and has the potential to inform future research and conservation efforts on habitat 9210*. The collaboration between robotic engineers and plant scientists provides a unique perspective on the forest ecosystem and underscores the potential for interdisciplinary work in this field. This dataset constitutes an important contribution to the ongoing effort to monitor and conserve habitats globally, particularly in light of the challenges posed by global changes.

## Background & Summary

Regularly monitoring habitats is becoming increasingly important for preserving biodiversity through evaluating the conservation status of the habitat itself and the attainment of conservation targets. Indeed, the Directive 92/43/EEC of the European Council (Habitats Directive^1^) requests that member countries periodically carry out such monitoring campaigns. It also established a network of critical habitats for rare and endangered species, including certain uncommon natural habitat types that are protected in their own right: the Natura 2000 network^2–4^. On land and at sea, it crosses all 27 member states of the European Union. The network’s goal is to protect the most priceless and imperiled species and habitats in Europe for the long run. As far as habitats of community interest are concerned, the need for extensive monitoring is urgent. This is especially true for forest habitats, which occupy over 50% of the surface of Special Areas of Conservation (SACs) within the Natura 2000 network^5^.

In this work, we focus on beech forests, which represent key habitats under the Natura 2000 network. Our study case is on beech forests located in the Apennine mountain chain (central Italy), considered the most diverse and species-rich in the European Union^6^. In particular, we studied habitat “9210(*) Apennine beech forests with *Taxus* and *Ilex*”, listed as of priority interest for conservation in the Annex I of the EU Habitats Directive. Anthropic pressures such as the intensification of sylvicultural management and climate change with increasing temperatures and drought are threatening the maintenance of such forests in a good conservation status in central Apennines^7–10^, as required by the Directive 92/43/EEC. For such reasons, there is the need to develop new and repeatable monitoring strategies that take account of the many indicators of the conservation status and naturalness of beech forests, such as their floristic composition, structural features like the dimensions of trees and the amount of dead wood, and the amount of litter, mosses, and lichens^5^. The complex and fundamental task of monitoring forest habitats of community interest requires a high level of botanical expertise and knowledge, plus the ability to move for hours in wild unstructured environments. Nowadays, this task can be carried out only by highly trained human operators. This process implies high costs in terms of economic and human resources for the EU Countries. In light of these considerations, the European Union project Natural Intelligence aims at improving human monitoring abilities through the introduction of robotic workforce. We use legged robots in particular to collect data on the habitat in a manner that closely resembles surveys done by humans.

The habitat 9210* includes thermophilous beech forests harbouring many endemics and characterized by a tree layer dominated by *Fagus sylvatica* and a herbaceous layer composed of nemoral species^10^. The assessment of the conservation status of this habitat can be performed through the measurement of parameters related to its structure and function, such as dendrometric data, floristic data, and identification of typical species^11–14^. Some key indicators of favourable conservation status of this habitat are herbaceous sciaphilous-nemoral species (i.e., geophytes of the genera *Anemonoides*, *Gagea*, *Corydalis*^15,16^;). For instance, the increase of shade-tolerant species (mostly geophytes) was reported for unmanaged and ancient forests^17,18^. On the other hand, early warning species indicating the occurrence of climate change phenomena, such as increasing temperatures and/or drought, as well as of the intensification of sylvicultural management, are considered as indicators of a non-favourable conservation status. In the Apennine sites of the habitat 9210*, typical examples of the latter case are the occurrence of meso-thermophilous species linked to woodlands of the hilly belt, such as *Cyclamen hederifolium*, *Primula vulgaris*, *Viola alba* subsp. *denhardtii*, and/or of species related to open habitats, such as *Brachypodium genuense*, *B. rupestre*, *Festuca* sp. pl., and *Sesleria* sp. pl.^16,19^. Several other indicators are needed to define the conservation status of the habitat (HD, art. 17^12^;), including vegetation cover, amount of bare soil, rocks, stones, presence/abundance of typical and early warning species, and structural data, such as tree diameter at breast height and tree height. These can be used as indicators of the ecological and dynamic differences between *F. sylvatica* forests and to evaluate the structure and functions of the habitat 9210*. The collection of field data is pivotal for the assessment of the conservation status of habitats.

**Fig. 1:**
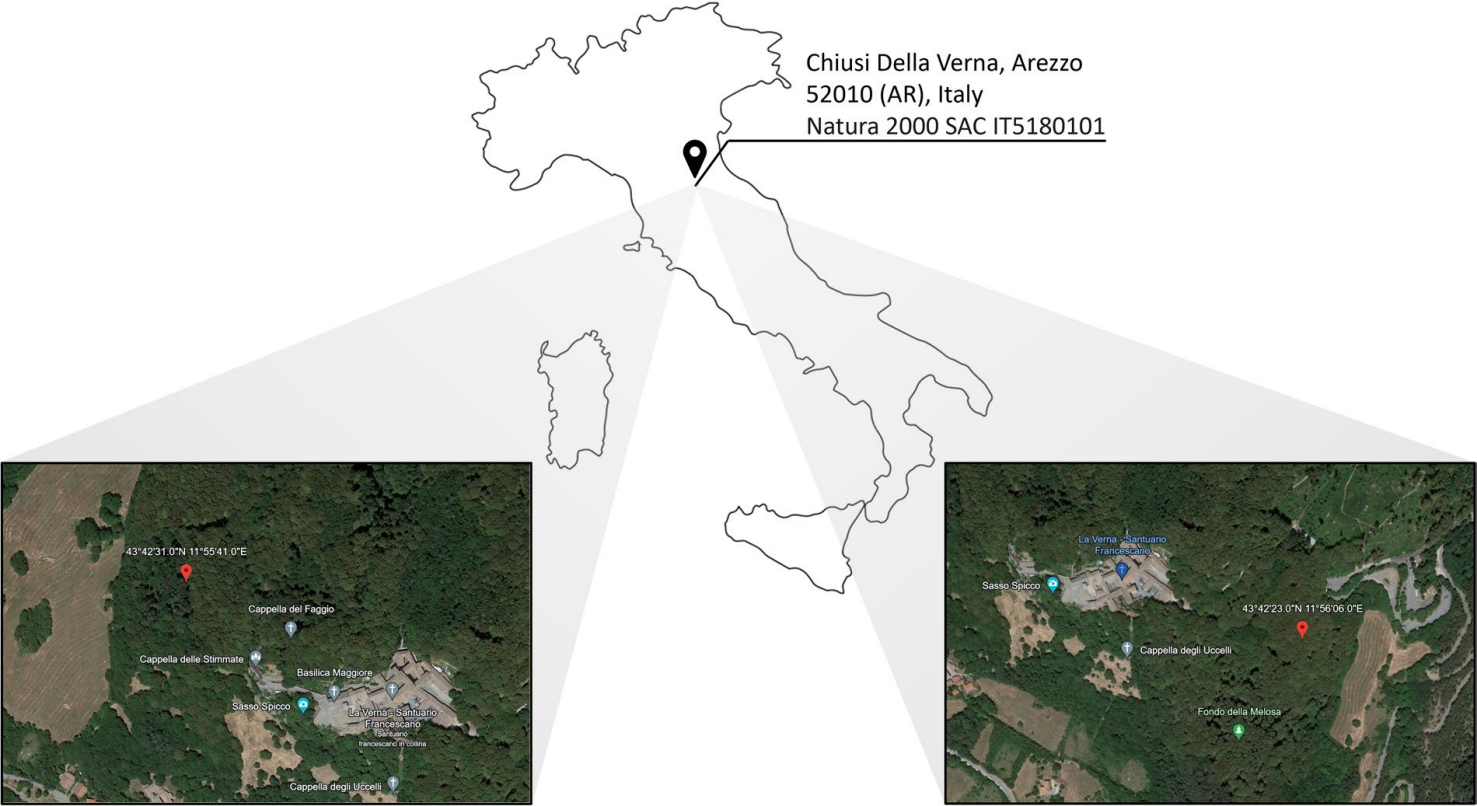
The location in which the data presented in this paper were collected: Chiusi della Verna, Tuscany, Itay. Two inserts depict the more precise locations.

**Fig. 2:**
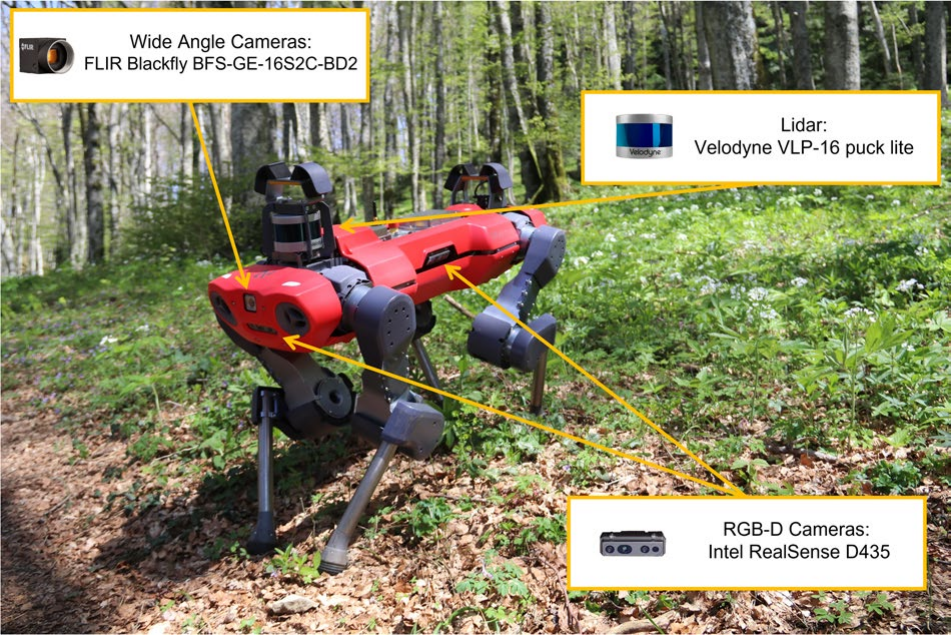
The robot ANYmal C in habitat 9210*. The sensors employed for data gathering are one Velodyne VLP-16 puck lite, two FLIR Blackfly BFS-GE-16S2C-BD2 wide angle cameras, and four Intel RealSense D435 RGB-D cameras.

In this data paper, we present the data gathered in a beech forest of Tuscany, central Italy (Fig. 1). This data has been collected using the quadrupedal robot ANYmal C^20^ (Fig. 2) by a group of plant experts and robotics engineers during habitat monitoring missions. The collected data is divided into four groups: (i) photos of indicator plant species of the forest habitat, which are either typical or early warning species, (ii) three-dimensional data about the monitored areas, (iii) photos, videos, and robot information during autonomous robotic monitoring missions, and (iv) during human-operated robotic monitoring missions. We have already presented a similar dataset for grassland habitat 6210* in^21^.

Researchers from a variety of disciplines can benefit from using this dataset because of its multidisciplinary scope. On the one hand, robotic engineers could, for instance, benchmark the performance of the robots and test or validate their own methods using the point clouds and the information about the robot state. On the other hand, botanists could evaluate the accuracy of this data as well as the habitat’s conditions using the plant videos and images that the robot captured, or computer scientists could test their AI algorithms for identifying and classifying different species using these data.

## Methods

We acquired the data in Chiusi della Verna, Tuscany, central Italy, on the 27th and the 28th of April, 2022. This location is within the Natura 2000 network, SAC IT5180101, La Verna-Monte Penna, within the National Park Foreste Casentinesi, Monte Falterona e Campigna (Fig. 1). The choice of the days was based on the blooming period of the studied indicator species, which is from March to May. A team of both robotic engineers and plant scientists employed the robot ANYmal C^20^ (Fig. 2), produced by ANYbotics AG, for data acquisition. This quadrupedal robot can collect information regarding the habitat using its on-board sensor while navigating the forest in both an autonomous or teleoperated way. Four Intel RealSense D435 RGB-D cameras (https://www.intelrealsense.com/depth-camera-d435/) are placed one per side of the robot body and can capture images in Full HD resolution and/or record videos of the same resolution with 30 fps. Additionally, a Velodyne VLP-16 puck lite LiDAR (https://velodynelidar.com/products/puck-lite/) is mounted on the rear-top part of the robot body allowing it to acquire a 3D map of the surrounding environment. ANYmal also mounts two wide angle FLIR Blackfly BFS-GE-16S2C-BD2 cameras. These, however, were not used in the present work for gathering data. Data concerning the state of the robot during habitat data harvesting are saved as ROS bag files through the dedicated ROS interface of the robot. Details about ROS bag files can be found on the official documentation (http://wiki.ros.org/rosbag). Plot position was recorded using a portable GPS (Garmin Colorado 300) to assist future relocation of the plot, consistently with European Habitat and vegetation monitoring protocols^11,22^. For each plot, georeferencing was performed with at least 10 m of positional accuracy, which is defined as the value of the radius of the circle of unknown around the true position. During our survey, the measured accuracy (10 m) was larger than the common values of GPS accuracy because of the presence of tree canopy in forest environments^23–25^. Although a cm-level accuracy would be more desirable to precisely relocate individual plots in repetitive surveys to obtain a network of permanent plots, our values of location accuracy suffice for most habitat and vegetation resurveying studies, e.g., quasi-permanent resurveys^26,27^.

Prior to field sampling, we identified in a GIS environment the polygons where the habitat 9210* covers 70% or more in the SAC, based on a detailed habitat map produced by the HaSCITu project (Habitat in the Sites of Conservation Interest in Tuscany^28^;). In such locations, data acquisition and assessment of the habitat conservation status are routinely carried out by trained botanists. Once in the field, we selected two suitable sites within the forest. Suitability was assessed based on the accessibility to both humans and the robot. This is because, despite the impressive mobility of the robot ANYmal C, safe locomotion is guaranteed only within some operational limits. An exhaustive description of these limits is out of the scope of this paper, however, we report a few examples: the slope of the terrain should not be greater than 30°, gaps in the terrain should not be bigger than 30 cm, and the maximum stepping height should be less than 25 cm. For more details on the robot’s operational limits, please refer to the producer website (https://www.anybotics.com/anymal-specifications-sheet/).

The dataset provided through this paper is comprised of four sets of data: (i) species data, (ii) 3D mapping data, (iii) autonomous monitoring mission data, and (iv) teleoperated monitoring mission data. The methods of acquisition of these are described in the following sections.

### Species data

This first part of the provided dataset is composed of photos of four different indicator species, which are divided into three typical species of the habitat 9210*^29^, and one early warning species. The typical species are *Anemonoides nemorosa* (L.) Holub (Ranunculaceae), *Anemonoides ranunculoides* (L.) Holub (Ranunculaceae), and *Corydalis cava* (L.) Schweigg. & Körte (Papaveraceae). The early warning species is *Primula vulgaris* Huds. (Primulaceae), an indicator of increasingly warmer and drier conditions in beech forests. Species nomenclature follows the World Flora Online portal^30^. In Table 1, we summarize the number of photos per species contained in the dataset.

**Table 1:** The four indicator species (family) of the habitat 9210* and the number of photos for each of them.

Name	Type	# Photos
*Anemonoides nemorosa* (L.) Holub (Ranunculaceae)	Typical species	101
*Anemonoides ranunculoides* (L.) Holub (Ranunculaceae)	Typical species	39
*Corydalis cava* (L.) Schweigg. & Körte (Papaveraceae)	Typical species	80
*Primula vulgaris* Huds. (Primulaceae)	Early warning species	39

All the selected plant species are herbs growing in the understory of mesic forests, and the three typical species are especially related to beech forests representing the habitat 9210*. They typically flower in early spring (March to May), before tree leaves are fully developed, to take advantage of the optimum of light availability in the forest habitat. *A. nemorosa* is a rhizomatous geophyte distributed in temperate to cold regions of Eurasia and North America. Flowers are single, with white petals and yellow anthers, and leaves are compound-palmate. *A. ranunculoides* is a rhizomatous geophyte as well, but its distribution is limited to Europe. Flowers are often single or rarely 2–5 per stem, with bright yellow petals and anthers and compound-palmate leaves. *C. cava* is a bulbous geophyte distributed in Europe. Flowers are gathered in raceme inflorescences including 5–50 elements, with white to purple petals. Leaves are pinnate-compound. *P. vulgaris* is a hemicryptophyte with a European distribution. Stemless, with yellow petals, and whole, spatulate leaves gathered in a basal rosette. Compared to the three typical species, *P. vulgaris* usually lives in many other types of mesic forests and their margins^31^ and is typical of hilly mesophilous woods with tree canopy dominated by *Carpinus betulus* and/or *Quercus cerris*^32^; in beech forests, it can be considered an indicator of xero-thermophilous conditions.

**Fig. 3:**
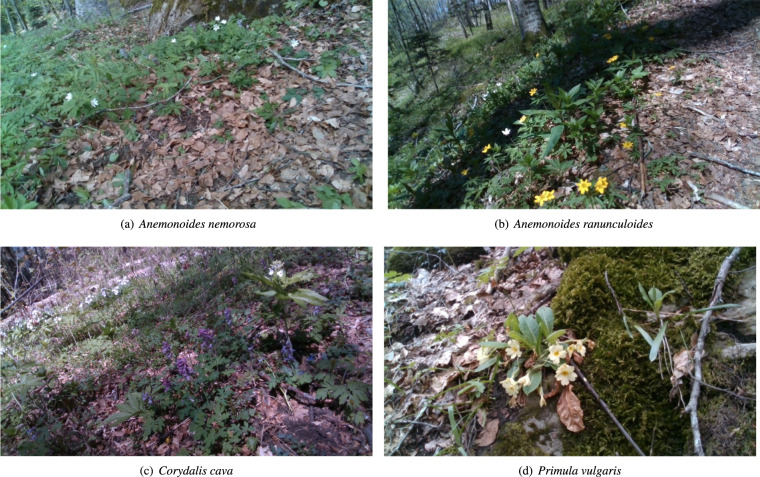
The four typical species of habitat 9210*. These pictures were taken by the on-board cameras of the robot.

The data acquisition was as follows: firstly, the plant scientists followed the guidelines in^31^ to identify the aforementioned indicator species. Subsequently, a roboticist guided the robot in teleoperated mode, stopping it facing the identified instance of species. Notice that one or more instances of the four indicator species may have been present in front of the robot at this time. Hence, a photo of a particular species contains at least one instance of it. Table 1 reports the number of photos per species contained in the provided dataset. These numbers roughly reflect the abundance of species in the chosen location within habitat 9210*. An example for each of the four indicator species mentioned before is shown in Fig. 3.

### 3D Mapping data

This second part of the data is made of three-dimensional spatial data provided in the form of point clouds, obtained using the LiDAR sensor. These describe, in dimension and shape, the environment surrounding the robot, while it is is teleoperated by a human. The robot is positioned in a randomly chosen area and GPS data, date, and weather information are recorded. These are relevant, also for the later described monitoring mission data, in order to fairly compare data against past and future surveys. Subsequently, the mapping procedure is started from ANYmal’s ROS Graphical User Interface. Then, the robot is teleoperated to locomote forward for 4 m, perform a 180° turn and return to the initial position. Finally, the mapping is stopped from the GUI and the point cloud data is saved. A video was recorded for each of the mapping procedures using the Canon Reflex camera in order to have a visual record. We also save information about the status of the robot during the mapping procedure using the ROS interface. Table 2 reports the ROS topics (http://wiki.ros.org/rostopic) recorded to store the information about the robot status. These are saved in ROS bag files (http://wiki.ros.org/rosbag). In particular, the status information about the robot comprises of its base position and velocity, joint positions, velocities, and accelerations, joint torques and currents, and the battery charge.

**Table 2:** The list of ROS topics from which we saved the data presented in this paper.

Topic Name	Description	M	A	T
/state_estimator/anymal_state	Robot info, e.g., base position [m] and orientation [rad], joint position [rad], velocity [rad/s], acceleration [rad/s^2^], and torque [Nm].	✓	✓	✓
/log/state/current/LF_HAA	Current [A] of the hip adduction/abduction joint of the left fore leg	✓	✓	✓
/log/state/current/LF_HFE	Current [A] of the hip flexion/extension joint of the left fore leg	✓	✓	✓
/log/state/current/LF_KFE	Current [A] of the knee flexion/extension joint of the left fore leg	✓	✓	✓
/log/state/current/LH_HAA	Current [A] of the hip adduction/abduction joint of the left hind leg	✓	✓	✓
/log/state/current/LH_HFE	Current [A] of the hip flexion/extension joint of the left hind leg	✓	✓	✓
/log/state/current/LH_KFE	Current [A] of the knee flexion/extension joint of the left hind leg	✓	✓	✓
/log/state/current/RF_HAA	Current [A] of the hip adduction/abduction joint of the right fore leg	✓	✓	✓
/log/state/current/RF_HFE	Current [A] of the hip flexion/extension joint of the right fore leg	✓	✓	✓
/log/state/current/RF_KFE	Current [A] of the knee flexion/extension joint of the right fore leg	✓	✓	✓
/log/state/current/RH_HAA	Current [A] of the hip adduction/abduction joint of the right hind leg	✓	✓	✓
/log/state/current/RH_HFE	Current [A] of the hip flexion/extension joint of the right hind leg	✓	✓	✓
/log/state/current/RH_KFE	Current [A] of the knee flexion/extension joint of the right hind leg	✓	✓	✓
/pdb/battery_state_ros	Battery information, e.g., charge percentage, voltage [V], current [A].	✓	✓	✓
/path_planning_and_following/navigate_to_goal/result	Info on the success (or failure) of the robot in reaching the desired navigation goal	✗	✓	✗
/path_planning_and_following/trajectory_poses	The planned Cartesian poses (waypoints) tracked by the robot to reach the goal	✗	✓	✗
/path_planning_and_following/active_path	The actual Cartesian path through the waypoints followed by the robot to reach the goal	✗	✓	✗
/tf	Coordinate frames and transformations between them (TFs) (http://wiki.ros.org/tf2)	✓	✓	✓

The last three columns specify if the topic has been saved during the mapping procedure (**M**), the autonomous mission (**A**), and the teleoperated mission (**T**).

In this data paper, we provide three mappings of locations in the forests of Chiusi Della Verna. Some details of these acquisitions are briefly described in the first three rows of Table 3.

**Table 3:** Date, time, weather information, and georeferencing information of the 3D mappings, the autonomous and the teleoperated monitoring missions.

Type	Name	Date	Time	Weather	Latitude	Longitude
3D Mapping	Mapping 1	27 April 2022	15:35	Sunny	43°42′23″N	11°56′06″E
	Mapping 2	27 April 2022	16:04	Sunny	43°42′23″N	11°56′06″E
	Mapping 3	28 April 2022	18:15	Sunny	43°42′31″N	11°55′41″E
Autonomous Monitoring	Mission 1	27 April 2022	16:21	Sunny	43°42′23″N	11°56′06″E
Teleoperated Monitoring	Mission 1	28 April 2022	18:36	Sunny	43°42′31″N	11°55′41″E

### Autonomous monitoring mission data

This third part of the dataset relates to the autonomous monitoring mission carried out using the robot in Chiusi Della Verna. Robotic monitoring was carried out in correspondence to the plots used by plant scientists for their sampling. Then, georeferencing data (GPS coordinates) and weather data are noted. As mentioned before, these enable rigorous comparisons with past or future monitoring missions. Additionally, weather data can also be used to estimate sun light.

**Fig. 4:**
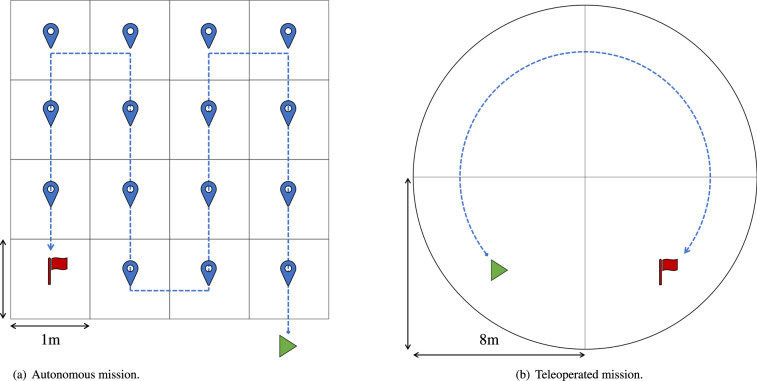
Motion of the robot during the (**a**) autonomous and (**b**) teleoperated monitoring missions. The robot starts from the green triangle and reaches the final goal (red flag) following the directed dashed blue path. The four cameras of the robot record the mission video and the robot status is saved in ROS bag files.

The autonomous monitoring makes use of a previously acquired 3D map of the environment to allow the robot to localize itself in the forest. In the case of the presented autonomous mission, a map acquired during the 3D mapping procedure described in the previous section is used. We do not record point cloud data during the execution of the mission since this would not provide valuable information with respect to the previous mapping. Then, the robot attempts to take detailed and close-up photos and videos of the flora at regular intervals. In order to do so, it moves, within an area of 4 × 4 m^2^, along waypoints on a zig-zag like path starting from the bottom right corner of the square towards the top left corner. See Fig. 4a for a visual representation of this path. The robot reaches each waypoint with the same initial orientation, it stops, and takes four photos (one per each of the four RGB-D cameras). The same four cameras also record videos of the whole mission. Additionally, ROS bags of the robot status information and external videos using the Canon Reflex camera are also saved. For this autonomous mission, Table 2 reports the ROS topics recorded to store not only the information about the robot status, but also the ones about reaching the navigation waypoints.

We report some more information about this monitoring mission on the fourth row of Table 3.

### Teleoperated monitoring mission data

The last part of the presented data regards the teleoperated mission. In this case, a circular plot with a radius of 8 m was placed. This plot shape is commonly used to survey forest plant communities, as it offers numerous benefits. It is easily established, it provides a straightforward means to determine whether a tree falls within the plot, and it contains fewer trees along its edges compared to other plot shapes with equivalent area^33^. In repetitive surveys or when used in remote sensing applications, the circular plot offers the operational convenience of needing the location of only its central point to be recorded^34^. The robot starts from the border of the circle. A roboticist guides the robot in teleoperated mode in a clockwise direction (see Fig. 4b) and stops the robot at regular intervals of one metre to take photos using the four on board cameras. This procedure continues until images of the whole area have been acquired. Moreover, the robot also records the whole video of the monitoring. Together with this, a ROS bag file saving the status of the robot and an external video are also recorded. Here, since the robot is moving in teleoperation, no mapping is required.

The last row of Table 3 reports additional information about this teleoperated mission. Similarly to the autonomous mission, Table 2 reports recorded ROS topics. These are the same as for the mapping procedures.

## Data Records

**Fig. 5:**
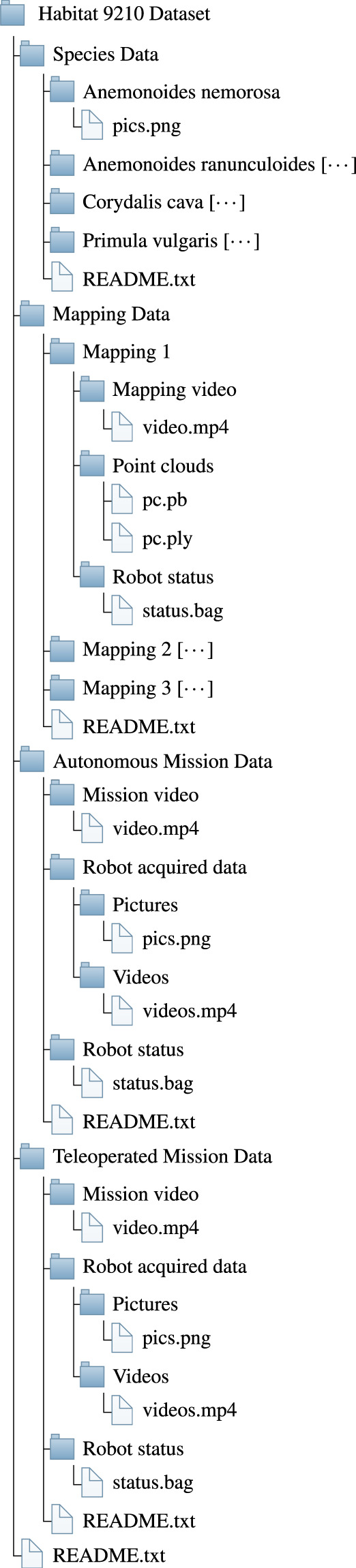
The directory structure tree of the dataset presented in this paper. The symbol [⋯] next to a folder name indicates that its subtree is similar to the previous same-level folder.

The data are available on Zenodo^35^ at 10.5281/zenodo.10013693. We also provide example code to extract and analyse the ROS bag files on Zenodo^36^ and GitHub^37^. The four sets of data that we provide are structured as in the tree in Fig. 5. Each of the four subtrees contains a README.txt file that describes the corresponding set of data. The first set is Species Data, and it comprises a folder for the pictures of the typical and early warning plant species found in the surveyed habitat 9210*. Table 1 reports the number of pictures for each of the four indicator species studied in this set of data.

The second set, 3D Mapping Data, is a collection of 3D mappings saved as point clouds and the associated videos. These are contained in two separate folders, namely Point Clouds and Mapping Videos.

The third and fourth sets, Autonomous and Teleoperated Monitoring Mission Data, contain all the information collected during the monitoring missions performed by the robot. Folders contain the recorded videos, the multimedia data from the robot, and robot status.

### Data formats

In the following sections, we provide some technical details about the file formats of the data.

#### .jpg

This standard image file format can be opened and viewed by commonly available image viewing software on all major operating systems. In this dataset, the pictures collected by the robot during the monitoring missions and the species data photos are in this particular jpg format.

#### .mp4 and .avi

These two standard video file formats can also be opened and watched by commonly available video players on all major operating systems. In this dataset, the videos recorded by the on-board cameras of the robot are in avi format, while the ones recorded using the Canon Reflex camera are in mp4 format.

#### .ply and .pb

Three-dimensional mapping data are stored as point clouds in two of the most used formats: ply and pb. The former is the so called Polygon File Format (ply). It is a standard file extension for 3D models that can be opened using 3D modelling software, e.g., MeshLab, or by more common math processing tools, such as MATLAB (https://www.mathworks.com/products/matlab.html). The latter format (pb) is, instead, the most wide-spread point cloud format within ROS, in particular in ANYmal research (https://www.anymal-research.org/). We provide both formats to ease users with handling 3D mappings.

#### .bag

**Fig. 6:**
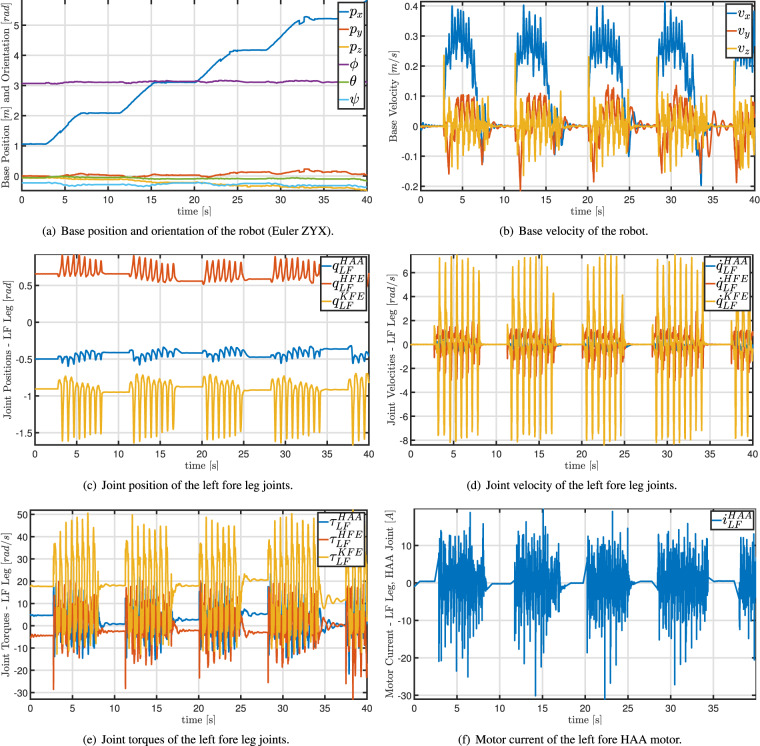
Plots of the extracted data from the ROS bag of the autonomous mission described in this paper (see folder *Autonomous Mission* in Fig. 5). Therein, the robot moved as shown in Fig. 4. Only an eighth of the entire mission (40 s out of 320 s) is plotted for the better image clarity.

Bag files are used in ROS for storing message data flowing in ROS topics (http://wiki.ros.org/rostopic). A list of the topics that were saved in the ROS bags provided herein is available in Table 2. These topics transmit the data and information read from the robot and, hence, the bag files save them in suitable structures. The website of ANYmal Research (https://www.anymal-research.org/) is to be referred for more details about the on board sensors and the data output from them through the ROS interface. Figure 6 shows some of these measured quantities during the above mentioned autonomous mission. Within ROS a variety of tools exist for extracting, visualizing and analysing the data stored in bag files. For researchers outside robotics, MATLAB (https://www.mathworks.com/products/matlab.html) could be a valid alternative to handle this type of files.

## Technical Validation

Both the team of robotic engineers — Franco Angelini (FA), Mathew Jose Pollayil (MJP), and Manolo Garabini (MG) — and plant scientists — Leopoldo de Simone (LDS), Emanuele Fanfarillo (EF), Tiberio Fiaschi (TF), Simona Maccherini (SM) and Claudia Angiolini (CA) — provided quality assurance during the fieldwork. Each author monitored the data collection and carefully checked the final dataset for errors and inconsistencies.

It is also important to note that the supplied data are provided in their raw form without any post-processing that can compromise their validity. A number of decisions that are specifically discussed in the following ensure the consistency of the data collection.

### Area selection

The area to be surveyed has been selected by plant scientists with a high degree of expertise on the local flora and vegetation. The forest in La Verna-Monte Penna (SAC IT5180101), within the National Park Foreste Casentinesi, Monte Falterona e Campigna in Chiusi della Verna, Arezzo 52010 (AR), Tuscany, Italy, is part of the Natura 2000 network, and it is a good example of the EU Annex I Habitat “9210(*) Apennine beech forests with *Taxus* and *Ilex*”.

### Time selection

The dates of the surveys, i.e., the 27th and the 28th of April 2022, were suggested by the plant scientists according to the ideal time to monitor the indicator species of habitat 9210* in their full bloom. This is because the period from March to May is known to be the blooming period of the studied indicator species.

### Species data validity

The selection of the indicator species for habitat 9210* has been carried out in accordance with the EU guidelines of international journals^30^, as mentioned in the Methods section. The photos of the plants were selected and attributed to species following the recommendations by plant scientists.

### Mapping data validity

The data saved by the robot during mapping is provided raw without any processing. Its validity is ensured by the absence of any localization issues signaled by the robot after this procedure since the saved map is used by ANYmal to localize itself in the environment using Simultaneous Localization and Mapping (SLAM) algorithm.

### Monitoring data validity

The validity of the data collected during monitoring missions is ensured by the methodologies used to conduct them, which are based on guidelines from manuals and scientific articles^11,38^. The robot carefully performs relevé by following the same procedures adopted by the plant scientists, hence, complying with these guidelines. Moreover, the robot signals possible failures in acquiring data. No such event occurred during the performed missions. Finally, since the data are provided without any modifications for analyses, no post-processing related corruption is possible.

### Database validity

The database has been produced successively to the completion of data collecting. We added only valid and non-corrupt data. Both teams meticulously revised each entry of the database to ensure its validity. The plant experts verified each image and video of the dataset, in particular the classification performed in the section “Species Data”. Similarly, the robotic engineering team double checked the point clouds and robot status data by inspecting them and running test scripts to ensure their legitimacy.

## Usage Notes

The usefulness of the provided data lies in its multidisciplinary scope. Both robotic and botanical research can benefit from the images and videos presented in this paper. For instance, machine learning techniques can be employed to detect and classify the flora; this can aid robotic locomotion in planning optimal paths and plant researchers to assess the health status of the species. All European Countries must carry out habitat monitoring campaigns every six years, and our dataset can be used to assess the effectiveness of conservation measures and the achievement of conservation targets. Moreover, the provided data will be useful for a wide range of researchers in botany, ecology, and forestry. For instance, potential uses are: (i) detailed mapping of the distribution of plant individuals; (ii) measurements of plant abundance with a high degree of accuracy and precision; (iii) assessment of the health status of the plants; (iv) measurements of plant morpho-functional traits; (v) recording of phenological observations (e.g., date of flowering and fruiting, abundance of flowers and fruits); (vi) calculation of wood biomass for both forestry and habitat conservation purposes; (vii) coupling of structural and vegetation data to explore the patterns of plant diversity in relation to forest structure; (viii) providing, through the structural data, a sound basis for orienting forest management strategies, such as identifying suitable stands to be promoted to natural evolution, for assessing forest conservation status or maximizing the productivity of different ecosystem services^6,39^.

Some more details about these example applications of the presented dataset might be of interest for the readers. For instance, measurements of plant abundance in an area can be carried out employing the photos collected by the robot in the same area. These can be duly labelled by expert botanists and used to train neural networks to identify plant species from photos, e.g.^40^, Such a network can be used to infer the percentage of presence of a given species in an area.

Another example is the automated measurement of forest structural parameters from three-dimensional forest point cloud data^41–43^: the mapping data provided by this paper comprises point clouds from which forest structural parameters such as tree number per stand and their diameter at breast height can be automatically extracted. The extraction of structural data may be of use in comparing forest communities in terms of diameter distribution and tree species composition^44–46^; its potential for classifying and monitoring European Union forest habitats and evaluating their conservation status was partially explored by^39^.

The interested reader can find more details about the examples of potential uses in the literature^38^.

### Template code for data extraction

We provide a template example of MATLAB script for ROS bag analysis in Code 1. This contains the basic lines of code to open a bag file and read its content within a specified topic. This example can be run in MATLAB 2022a (https://www.mathworks.com/products/matlab.html) or later together with ROS toolbox (https://www.mathworks.com/products/ros.html). The topics that can be set to extract data from the bags are reported in Table 2. These topics and the details about the sensors employed on ANYmal C to record data are described in detail on the ANYmal Research Website (https://www.anymal-research.org/). However, a research partnership with ANYbotics is needed to access this.

#### Code 1

Template MATLAB code for extracting data from ROS bag files.

It behooves us to highlight that the data presented in this paper is free to access independently by accessing ANYmal Research. Moreover, the template code shown in Code 1 and the code attached to this submission are enough to extract, visualize, and analyze the provided data.

## Data Availability

The GitHub page of the Research Center E. Piaggio^37^ and Zenodo^36^ host the MATLAB code attached to the data presented in this paper. This can be used to extract and visualize data from ROS bag files. In particular, the GitHub repository contains a README file describing each of the scripts individually.
